# Male Disadvantage in Oxidative Stress-Associated Complications of Prematurity: A Systematic Review, Meta-Analysis and Meta-Regression

**DOI:** 10.3390/antiox10091490

**Published:** 2021-09-18

**Authors:** Elke van Westering-Kroon, Maurice J Huizing, Eduardo Villamor-Martínez, Eduardo Villamor

**Affiliations:** 1 Department of Pediatrics, Maastricht University Medical Center (MUMC+), School for Oncology and Developmental Biology (GROW), 6229HA Maastricht, The Netherlands; elke.kroon@mumc.nl (E.v.W.-K.); m.huizing@mumc.nl (M.J.H.); e.villamorm@gmail.com (E.V.-M.); 2 Statistics Netherlands, 6412HX Heerlen, The Netherlands

**Keywords:** preterm birth, oxidative stress, sex differences, male disadvantage, female advantage, bronchopulmonary dysplasia, retinopathy of prematurity, necrotizing enterocolitis, intraventricular hemorrhage, periventricular leukomalacia, mortality

## Abstract

A widely accepted concept is that boys are more susceptible than girls to oxidative stress-related complications of prematurity, including bronchopulmonary dysplasia (BPD), retinopathy of prematurity (ROP), necrotizing enterocolitis (NEC), intraventricular hemorrhage (IVH), and periventricular leukomalacia (PVL). We aimed to quantify the effect size of this male disadvantage by performing a systematic review and meta-analysis of cohort studies exploring the association between sex and complications of prematurity. Risk ratios (RRs) and 95% CIs were calculated by a random-effects model. Of 1365 potentially relevant studies, 41 met the inclusion criteria (625,680 infants). Male sex was associated with decreased risk of hypertensive disorders of pregnancy, fetal distress, and C-section, but increased risk of low Apgar score, intubation at birth, respiratory distress, surfactant use, pneumothorax, postnatal steroids, late onset sepsis, any NEC, NEC > stage 1 (RR 1.12, CI 1.06–1.18), any IVH, severe IVH (RR 1.28, CI 1.22–1.34), severe IVH or PVL, any BPD, moderate/severe BPD (RR 1.23, CI 1.18–1.27), severe ROP (RR 1.14, CI 1.07–1.22), and mortality (RR 1.23, CI 1.16–1.30). In conclusion, preterm boys have higher clinical instability and greater need for invasive interventions than preterm girls. This leads to a male disadvantage in mortality and short-term complications of prematurity.

## 1. Introduction

Preterm birth is defined as birth before 37 completed weeks of gestational age (GA) and is further subdivided in extremely (GA < 28 weeks), very (GA 28 to <32 weeks), moderate (GA 32 to <34 weeks), and late (GA 34 to <37 weeks) preterm birth. Prematurity, particularly in the in the lowest ranges of GA, is a leading cause of infant mortality, as well as long-term morbidity [1]. 

Two widely accepted concepts in neonatal medicine are the so-called “male disadvantage” and “oxygen radical disease in neonatology”. The first concept is supported by a large body of evidence showing that boys are more susceptible than girls to adverse outcomes of prematurity, including bronchopulmonary dysplasia (BPD), retinopathy of prematurity (ROP), necrotizing enterocolitis (NEC), intraventricular hemorrhage (IVH), periventricular leukomalacia (PVL), chronic neurodevelopmental and cognitive impairment, and death [2,3,4,5,6,7]. 

The term “oxygen radical disease in neonatology” was coined by Saugstad in the 1980s when he hypothesized that complications of prematurity, such as BPD, ROP, NEC, IVH, or PVL, are different facets of one disease sharing a basic pathogenetic mechanism: increased oxidative stress and reduced endogenous antioxidant defenses [8,9]. Interestingly, preterm girls have higher antioxidant enzyme activity than preterm boys and it has been suggested that these differences may play a key role in the male disadvantage of prematurity [10,11,12].

Although the notion of male disadvantage of prematurity is more than five decades old, only sex-associated differences in mortality have been systematically reviewed [13]. Our current aim is to conduct a systematic review and meta-analysis on male-female differences in risk of developing oxidative stress-associated complications of prematurity. In addition to outcomes such as BPD, ROP, NEC, IVH, or PVL, we also analyzed potential male–female differences in obstetrical characteristics and clinical conditions in the first weeks of postnatal life. Finally, since it has been suggested that the male disadvantage of prematurity has undergone changes in the last few years [14], we investigated by meta-regression the influence of time and other variables on the association between infant sex and complications of prematurity.

## 2. Materials and Methods

The study was performed and reported according to the preferred reporting items for systematic reviews and meta-analyses (PRISMA) and meta-analysis of observational studies in epidemiology (MOOSE) guidelines [15]. Review protocol was registered in the PROSPERO international register of systematic reviews (ID = CRD42018095509). The research question was “Do preterm boys have a higher risk of developing short-term complications of prematurity than preterm girls?” 

### 2.1. Sources and Search Strategy

A comprehensive literature search was undertaken using the PubMed and EMBASE databases. The search strategy is detailed in Table S1. No language limit was applied. The literature search was updated up to February 2021. Narrative reviews, systematic reviews, case reports, letters, editorials, and commentaries were excluded, but read to identify potential additional studies. Additional strategies to identify studies included manual review of reference lists from key articles that fulfilled our eligibility criteria, use of “related articles” feature in PubMed, and use of the “cited by” tool in Web of Science and Google scholar.

### 2.2. Study Selection 

Studies were included if they had a prospective or retrospective cohort design, examined preterm (GA < 37 weeks) infants, and reported primary data that could be used to measure the association between infant sex and short-term complications of prematurity. We only selected studies in which infant sex was the independent variable and the perinatal characteristics and outcomes were the dependent variables. Studies that exclusively included late preterm infants (GA ≥ 34 weeks), or combined preterm and term infants were excluded. To identify relevant studies, two reviewers (E.V., E.V.-M.) independently screened the results of the searches and applied inclusion criteria using a structured form. Discrepancies were resolved through discussion or consultation with a third reviewer (M.J.H.). 

### 2.3. Data Extraction and Quality Assessment

Two reviewers (E.V., E.V.-M.) extracted data from relevant studies using a predetermined data extraction form, and two reviewers (E.W.-K., M.J.H.) checked data extraction for accuracy and completeness. Discrepancies were resolved by consulting the primary report. Data extracted from each study included citation information, language of publication, study design, location and frame time, patient characteristics, and results (including raw numbers or summary statistics when raw numbers were not available). Data were extracted for all obstetric and perinatal variables as well as clinical conditions and outcomes reported in each study.

Methodological quality was assessed using the Newcastle-Ottawa Scale (NOS) for cohort studies [15]. This scale assigns a maximum of 9 points (4 for selection, 2 for comparability, and 3 for outcome). NOS scores ≥ 7 were considered high-quality studies (low risk of bias), and scores of 5 to 6 denoted moderate quality (moderate risk of bias) [15].

### 2.4. Statistical Analysis

Meta-analysis was performed when at least three studies were identified that reported on the same variable or outcome measure. Studies were combined and analyzed using comprehensive meta-analysis V3.0 software (Biostat Inc., Englewood, NJ, USA). Due to anticipated heterogeneity, summary statistics were calculated with a random-effects model. This model accounts for variability between studies as well as within studies. For dichotomous outcomes, the risk ratio (RR) with 95% confidence interval (CI) was calculated. For continuous outcomes (example: GA), the mean difference (MD) with 95% CI was calculated. Statistical heterogeneity was assessed by Cochran’s *Q* statistic and by the *I*^2^ statistic. Potential sources of heterogeneity were assessed through subgroup analysis and/or random effects (method of moments) univariate meta-regression analysis as previously described [16,17]. For both categorical and continuous covariates, the R^2^ analog, defined as the total between-study variance explained by the moderator, was calculated based on the meta-regression matrix. Predefined sources of heterogeneity included the following characteristics of cohorts: mean or median GA, median year of birth, and geographical location (continent). We used the Egger’s regression test and funnel plots to assess publication bias. Subgroup analyses, meta-regression, and publication bias assessment were performed only for the main outcomes (BPD, IVH, PVL, ROP, NEC, and mortality) and when there were at least ten studies in the meta-analysis. A probability value of less than 0.05 (0.10 for heterogeneity) was considered statistically significant.

## 3. Results

### 3.1. Description of Studies and Quality Assessment

The flow diagram of the search process is shown in Figure S1. Of 1365 potentially relevant studies, 41 (including 625,680 infants, 319,470 males) were included [2,3,4,5,14,18,19,20,21,22,23,24,25,26,27,28,29,30,31,32,33,34,35,36,37,38,39,40,41,42,43,44,45,46,47,48,49,50,51,52,53]. Their characteristics are summarized in Table S2. The percentage of males in the cohorts ranged from 41.2% [32] to 66.2% [20] with a pooled percentage of 52.2% (95% CI 51.4 to 53.0). Four studies included exclusively twin infants [24,35,44,51] and two studies included exclusively singleton infants [43,49]. The quality score of each study according to the Newcastle-Ottawa Scale is depicted in Table S2. All studies received at least seven points, indicating a low risk of bias.

#### 3.1.1. Meta-Analysis

The following variables were reported in more than two studies and were therefore included in the meta-analysis: chorioamnionitis, hypertensive disorders of pregnancy, maternal diabetes, prenatal care, premature rupture of membranes, prolonged rupture of membranes, antepartum hemorrhage, antenatal corticosteroids, fetal distress, cesarean-section, birth in a non-tertiary hospital (outborn), 5′ Apgar score < 3, 5′ Apgar score < 7, intubation at birth, resuscitation at birth, birth weight (BW) below the 10th percentile, BW below the 3rd percentile or -2SD, early onset (<72 h) sepsis, late onset (>72 h) sepsis, undefined onset sepsis, hypotension, patent ductus arteriosus (PDA), respiratory distress syndrome (RDS), administration of surfactant, mechanical ventilation, pneumothorax, postnatal steroids, any BPD (defined as oxygen requirement on postnatal day 28), moderate/severe BPD (defined as oxygen requirement at the postmenstrual age of 36 weeks), any IVH (grade 1–4), severe IVH (grade 3–4), PVL, severe IVH or PVL, any ROP, severe ROP (stage ≥ 3 or requiring treatment), any NEC, NEC ≥ Stage II, and mortality before discharge.

**Figure 1:**
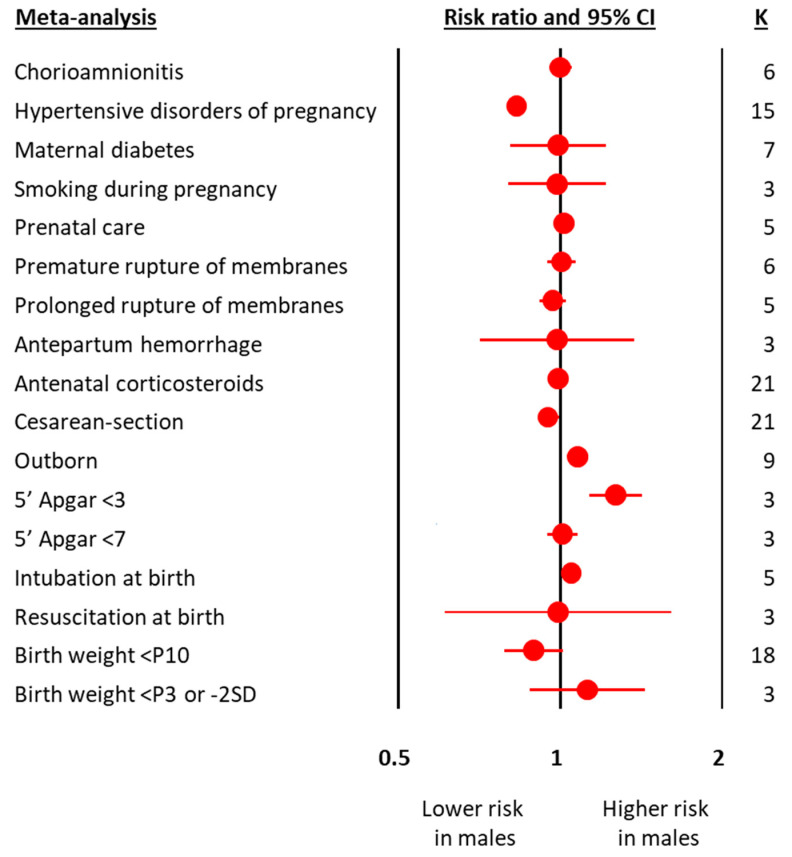
Summary of meta-analyses on the association between obstetric and perinatal characteristics of preterm infants and male sex. CI: confidence interval; K: number of studies; P3: 3rd percentile; P10: 10th percentile; SD: standard deviation.

**Figure 2:**
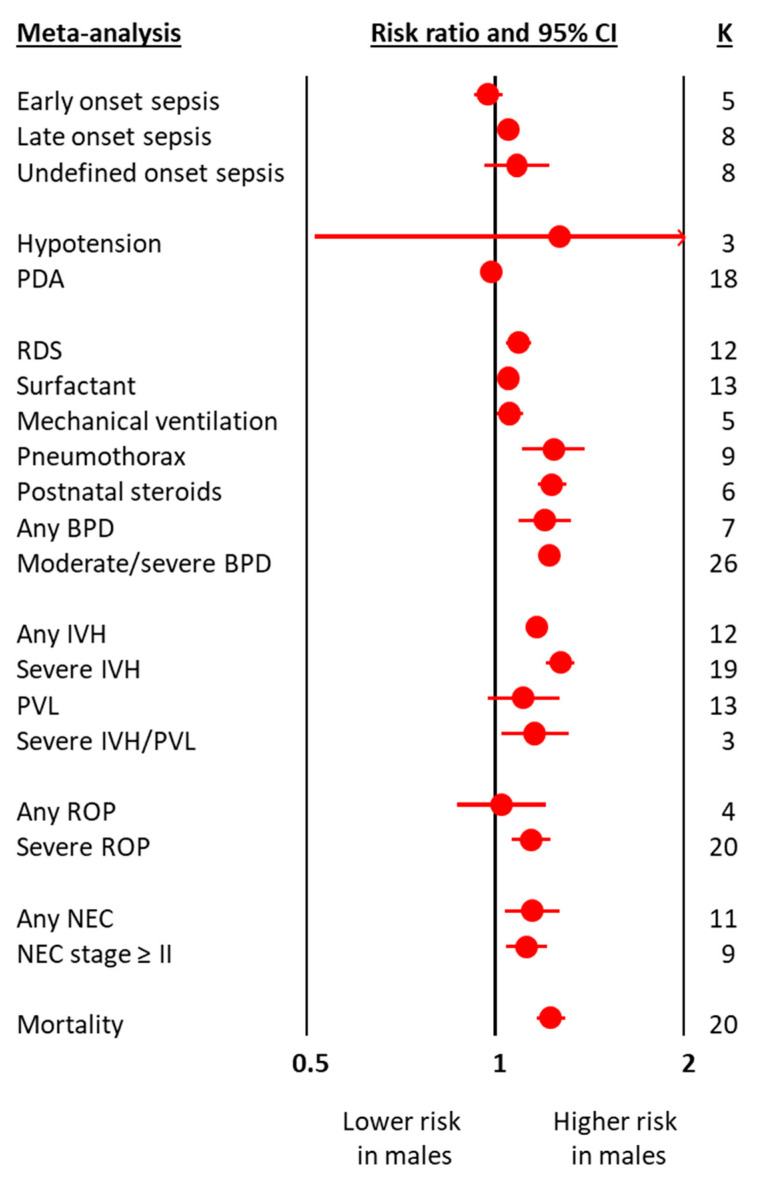
Summary of meta-analyses on the association between clinical characteristics and outcomes of preterm infants and male sex. BPD: bronchopulmonary dysplasia; CI: confidence interval; IVH: intraventricular hemorrhage; K: number of studies; NEC: necrotizing enterocolitis; PDA: patent ductus arteriosus; PVL: periventricular leukomalacia; ROP: retinopathy of prematurity.

The meta-analyses on obstetric and perinatal characteristics are summarized in Figure 1 and Table 1. The meta-analyses on clinical characteristics and outcomes are summarized in Figure 2 and Table 2. The individual meta-analyses for each outcome (BPD, IVH, PVL, ROP, NEC, and mortality) are shown in Figures S2–S12. Male sex was associated with a decreased risk of hypertensive disorders of pregnancy, fetal distress, and cesarean-section, but an increased risk of birth in a non-tertiary hospital, 5′ Apgar score < 3, intubation at birth, respiratory distress syndrome, surfactant use, pneumothorax, postnatal steroids, late onset sepsis, any BPD (Figure 2 and Figure S2), moderate/severe BPD (Figure 2 and Figure S3), any IVH (Figure 2 and Figure S4), severe IVH (Figure 2 and Figure S5), severe IVH or PVL (Figure 2 and Figure S7), severe ROP (Figure 2 and Figure S9), any NEC (Figure 2 and Figure S10), NEC ≥ stage II (Figure 2 and Figure S11), and mortality (Figure 2 and Figure S12). With regard to the continuous variables, BW was significantly higher in boys than in girls (Table 1). In contrast, no differences were found by infant sex in either GA or maternal age (Table 1).

Neither visual inspection of funnel plots (Figure S13) nor Egger’s test suggested publication or selection bias for any of the eligible meta-analyses (i.e., with at least ten studies). 

#### 3.1.2. Subgroup Analysis and Meta-Regression

Subgroup analysis based on the geographic location (continent) of the studies showed no significant differences for any of the outcomes analyzed, with only the exception of PVL (Table S2). The effect size of the association between male sex and PVL was significantly lower (meta-regression *p* = 0.048, R^2^-analog = 0.5) in the cohorts from America when compared with Asian and European cohorts (Table S3). 

**Figure 3:**
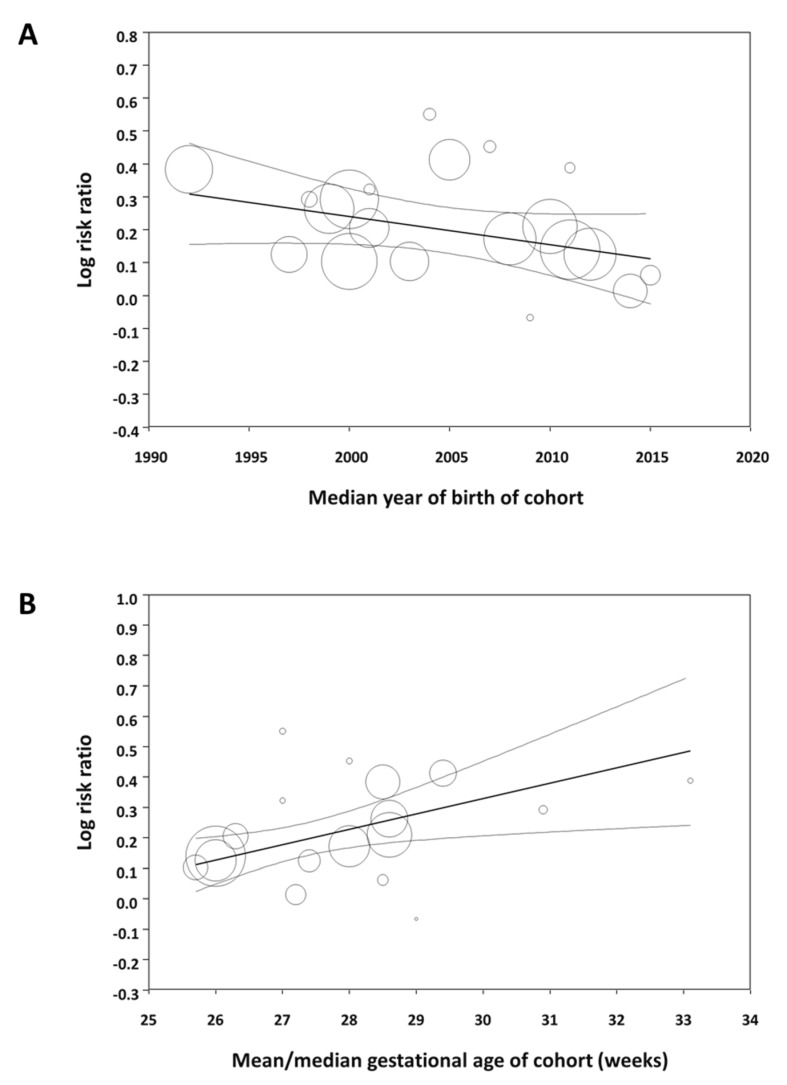
Meta-regression. (**A**) Plot showing the correlation between the association of male sex with mortality in preterm infants and the median year of birth of each cohort. A total of 24 studies were included (coefficient, −0.009; standard error, 0.004; *p* = 0.019; R^2^-analog, 0.37). (**B**) Plot showing the correlation between the association of male sex with mortality in preterm infants and mean/median gestational age of each cohort. A total of 18 studies were included (coefficient, 0.051; standard error, 0.014; *p* < 0.001; R^2^-analog, 0.75).

Meta-regression showed that the effect size of the association between male sex and mortality significantly decreased as the median year of the cohort increased (Figure 3A). In contrast, the association between male sex and the other outcomes did not correlate with the median year of birth of the cohort (Table S4). Meta-regression also showed that the effect size of the association between male sex and mortality significantly increased as the mean/median gestational age of the cohort increased (Figure 3B). The association between sex and the other outcomes did not correlate with the mean/median gestational age of the cohort (Table S4).

## 4. Discussion

To the best of our knowledge, this is the first systematic review and meta-analysis focused on male–female differences in short-term outcomes of prematurity. Our results confirm the presence of male disadvantage in mortality as well as relevant morbidities, including IVH, BPD, ROP, and NEC. Although the observed increases in risk are modest, they hold important implications for understanding preterm birth complications. Besides the short-term complications, we investigated whether other prognostic factors such as GA, birth weight, obstetric history, or clinical condition in the first days of life were different between boys and girls. We found no male–female differences in GA but the well-known difference is birth weight. In addition, meta-analysis showed that male sex was associated with decreased risk of being exposed to hypertension during pregnancy, developing fetal distress, and being born by cesarean section but increased risk of birth in a non-tertiary hospital, low Apgar score, intubation at birth, developing respiratory distress, being treated with surfactant and mechanical ventilation, developing pneumothorax, receiving postnatal steroids, and developing late onset sepsis. These differences in clinical course may have a major influence on the development of the pulmonary, neurological, ocular, and gastrointestinal complications of prematurity. On the other hand, our meta-analysis could not demonstrate that the rates of hypotension, PDA, or early onset sepsis were significantly different between boys and girls. 

Male–female differences in human health and disease have been recognized for many years [54,55,56,57]. In reproductive and perinatal medicine, there are numerous studies dealing with sex differences, extending from fertilization and embryo implantation to the neonatal period [58,59,60,61,62,63,64,65,66]. The male to female ratio at birth is generally estimated to be around 1.05–1.06 [63,64,65,67]. This excess of males at birth is known for centuries and has been extensively studied by demographers, statisticians, epidemiologists, and biologists [63]. Focusing exclusively on preterm birth, the excess of males is even higher with male to female ratios around 1.2 [63,64,65,68,69]. However, the underlying mechanisms for this difference remain unclear, with suggestions including sexual dimorphism in embryonic and fetal homeostasis, as well as in the pathophysiological pathways that trigger preterm birth.

Preterm birth is always the result of a pathologic process, which may not only contribute to early delivery but may also adversely affect neonatal outcomes [16,70,71,72,73]. The pathophysiological pathways, or endotypes, leading to very and extreme preterm birth are divided into two main groups: (1) intrauterine infection/inflammation, and (2) dysfunctional placentation [16,70,71,72,73]. The first group is related to chorioamnionitis and placental microbial invasion and is associated with preterm labor, pre-labor premature rupture of membranes, placental abruption, and cervical insufficiency. The second group is associated with hypertensive disorders of pregnancy (including preeclampsia, eclampsia, and pregnancy-induced hypertension), and the entity identified as fetal indication/IUGR [16,70,71,72,73]. The association between fetal sex and prematurity endotype has been particularly examined in the case of the dysfunctional placentation endotype. A number of meta-analysis showed that preterm preeclampsia is associated with carrying a female fetus, while pregnancies with a male fetus are associated with developing term and post-term preeclampsia [61,62]. Accordingly, we observed an increased risk of hypertensive disorders of pregnancy and fetal distress associated with female sex. In contrast, there was no evidence of sexual dimorphism for conditions related to the infectious-inflammatory endotype, such as chorioamnionitis or rupture of membranes. Nevertheless, it should be taken into account that the low number of studies reporting on these prenatal conditions limits the power of the meta-analysis to detect possible differences. In fact, it has been suggested that pregnancy with a male fetus may favor a more pro-inflammatory intra-uterine environment, leading to a higher incidence of infection/inflammation-driven preterm birth [59,69,74]. Our group is currently conducting a meta-analysis exclusively focused on the association between fetal sex and endotype of prematurity. 

Regardless of the imbalanced sex ratio at birth, the underlying mechanisms specifically responsible for the observed increase in neonatal morbidity in preterm boys are likely multifactorial and not yet fully elucidated. Possible explanations include male–female differences in mother–fetus interaction, rate of fetal development, molecular differences between sex chromosomes, epimutations that preferentially affect one sex, variations in antioxidant capacity, and hormonal differences [7,11,58,59,60,64,65,66]. Since the dawn of neonatology, the degree of pulmonary maturity at birth has been recognized as the critical factor in determining the survival and prognosis of preterm infants [75]. It was also noted early on that preterm boys had a higher rate and severity of respiratory distress syndrome than preterm girls [76]. This has been linked to the influence of sex hormones on lung development and maturation and to anatomic differences in lung development during fetal life [57,77,78]. As reviewed by Seaborn et al., the lung of male fetuses is exposed to higher levels of testosterone in the period preceding the surge of surfactant production [77,78]. Thereafter, both sexes are exposed to increasing levels of estradiol but the fetal lung has the capability to synthesize and inactivate sex hormones, and hence to modulate their action in a sex-dependent way [78]. The present meta-analysis confirms that the respiratory clinical course is less favorable in preterm boys than in preterm girls. As mentioned above, these differences in the first days of life may be critical for the later development of other complications of prematurity.

As mentioned in the introduction, oxidative stress plays a central pathogenic role in the development of most of the complications of prematurity [9,79,80]. Thus, sex differences in the development of antioxidant defenses are frequently pointed out as the key factor for male disadvantage among preterm infants [10,11,12]. From this perspective, the glutathione pathway is the most extensively studied [10,11,12]. Glutathione is the major endogenous soluble antioxidant in mammalian cells and its metabolism controls the intracellular levels of peroxides (via glutathione peroxidase), aldehydes (via glutathione S-transferase), and even radicals (via regeneration of oxidized vitamins C and E) [12]. As reviewed by Lavoie and Tremblay, numerous factors related to glutathione metabolism, including glutathione levels, activity of enzymes (glutathione peroxidase, glutathione reductase, glutathione S-transferase), and cellular uptake of cysteine have been found as sex-dependent in the placenta, umbilical cord, and blood cells of preterm infants [12]. Therefore, it has been suggested that antioxidant strategies in preterm infants should mainly target glutathione metabolism and be personalized considering, among others, the sex specificity [12]. 

The study cohorts included in our meta-analysis spanned a 30-year period (1986–2016) and some of the factors affecting outcome in the early 1990s may not be so relevant to current preterm populations. Moreover, it has been suggested that the advances in perinatal medicine, which have led to a decline in mortality and improved short-term outcomes for the most vulnerable preterm infants, have had a greater impact on boys than on girls [14]. Therefore, the male disadvantage of prematurity might be decreasing over the years [14]. We have tested this hypothesis by meta-regression and found that the male disadvantage in mortality among preterm infants tends to decrease as the cohorts include infants born in recent years. However, the increased risk of developing BPD, ROP, or NEC in males did not show this decreasing trend over the years. We also used meta-regression to analyze whether male disadvantage correlated with the gestational age of the cohort. Again, this meta-regression was only significant for mortality (Figure 3B). The association between male sex and risk of mortality decreased as the cohort had a lower mean gestational age. This effect of gestational age was not observed for any of the other complications of prematurity.

The major strength of our meta-analysis is the comprehensive database search to identify all the potential studies. Thus, the 41 included studies encompassed a total population of 625,680 infants from 16 different countries, providing a significant international representation. When we performed subgroup analyses based on continent, the only outcome where we found a significant geographic difference in sex ratio was PVL. It should be noted that, in contrast to the other outcomes analyzed, PVL was the only main complication of prematurity for which the meta-analysis did not show an increased risk associated with male sex. Subgroup analysis showed that the absence of male disadvantage for PVL was due to the marked differences between the American and the Asian and European cohorts (Table S2). Nevertheless, this finding may be an artifact due to the limited number of studies and therefore needs to be investigated in meta-analyses specifically focused on PVL. For all other outcomes, including IVH, BPD, ROP, NEC, and mortality, sub-group analysis did not show geographic differences, suggesting that the male disadvantage of prematurity is a ubiquitous phenomenon. 

The main limitation of our systematic review is that studies were included only if sex was the independent variable and the association between sex and outcome was reported for more than one complication of prematurity. Although this design allowed for comparing the impact of male disadvantage on the different outcomes, we excluded a large number of studies in which an individual outcome was the independent variable and sex, among other potential risk factors, was the dependent variable. Our group is now analyzing these studies separately. The results of these meta-analyses, which for outcomes such as ROP or BPD include more than 250 studies, will confirm the present findings and analyze more comprehensively the influence of factors such as changes in trends over the years or geographic location on male disadvantage.

## 5. Conclusions

The present data suggest that the clinical course of preterm males is more complicated than that of females from the earliest moments of life. This higher clinical instability in males seems particularly to affect the respiratory system and leads to higher mortality and short-term morbidity. Complications such as BPD, ROP, NEC, IVH, or PVL will have a serious impact on post-discharge growth and neurodevelopment, extending the male disadvantage to the years of childhood and adolescence. In numerous studies on health conditions and neurocognitive outcomes of former preterm infants, adult females frequently perform better than adult males [1,7,81,82,83]. An improved understanding of sex-specific requirements of preterm infants may lead to optimized strategies to avoid the sequelae of early life oxidative stress and inflammation [7].

## Figures and Tables

**Table 1:** Meta-analyses on the association between obstetric and perinatal characteristics of preterm infants and male sex.

Meta-Analysis	K	RR	95% CI		*p*	Heterogeneity	
			Lower Limit	Upper Limit		I^2^ (%)	*p*
Chorioamnionitis	6	1.001	0.953	1.052	0.969	67.3	0.009
Hypertensive disorders of pregnancy	15	0.829	0.803	0.856	<0.001	22.9	0.200
Maternal diabetes	7	0.991	0.808	1.214	0.927	65.9	0.007
Smoking during pregnancy	3	0.987	0.800	1.218	0.901	3.9	<0.001
Prenatal care	5	1.017	0.982	1.053	0.352	96.2	0.353
Premature rupture of membranes	6	1.006	0.947	1.068	0.852	62.8	0.020
Prolonged rupture of membranes	5	0.968	0.914	1.026	0.275	0.0	0.972
Antepartum hemorrhage	3	0.986	0.708	1.374	0.936	96.9	<0.001
Antenatal corticosteroids	21	0.992	0.982	1.003	0.143	44.9	0.012
Fetal distress	3	0.784	0.678	0.907	0.001	0.0	0.741
Cesarean-section	21	0.980	0.966	0.995	0.008	51.5	0.003
Outborn	9	1.077	1.027	1.128	0.002	0.0	0.682
Apgar 5′ <3	3	1.269	1.132	1.422	<0.001	0.0	0.726
Apgar 5′ <7	3	1.010	0.946	1.077	0.772	85.2	0.001
Intubation at birth	5	1.038	1.006	1.071	0.019	66.4	0.018
Resuscitation at birth	3	0.990	0.609	1.609	0.968	93.2	<0.001
Birth weight <P10	18	0.892	0.785	1.014	0.080	80.9	<0.001
Birth weight <P3	3	1.123	0.877	1.438	0.358	51.5	0.127
**Continuous variables**		**MD**					
Gestational age (weeks)	24	−0.10	−0.21	0.01	0.076	87.0	<0.001
Birth weight (g)	24	47.8	34.1	61.5	<0.001	91.5	<0.001
Maternal age (years)	10	0.0	−0.5	0.5	0.999	92.5	<0.001

Random effects analysis. Risk ratio (RR) > 1 indicates association of male sex with increased risk of the variable and RR < 1 indicates association of male sex with decreased risk of the variable. K: number of studies, MD: difference of means.

**Table 2:** Meta-analyses on the association between clinical characteristics and outcomes of preterm infants and male sex.

Meta-Analysis	K	RR	95% CI		*p*	Heterogeneity	
			Lower Limit	Upper Limit		I^2^ (%)	*p*
Early onset sepsis	5	0.975	0.924	1.030	0.371	0.0	0.459
Late onset sepsis	8	1.051	1.026	1.077	<0.001	17.0	0.296
Undefined onset sepsis	8	1.083	0.962	1.218	0.186	20.6	0.266
Hypotension	3	1.270	0.514	3.140	0.605	72.7	0.026
PDA	18	0.985	0.958	1.012	0.262	52.5	0.004
RDS	12	1.090	1.042	1.140	<0.001	96.1	<0.001
Surfactant	13	1.031	1.026	1.036	<0.001	41.4	0.059
Mechanical ventilation	5	1.054	1.003	1.108	0.038	54.7	0.066
Pneumothorax	9	1.240	1.104	1.393	<0.001	42.2	0.086
Postnatal steroids	6	1.234	1.169	1.302	<0.001	37.6	0.433
Any BPD	7	1.200	1.091	1.319	<0.001	66.0	0.004
Moderate/severe BPD	26	1.219	1.176	1.264	<0.001	71.4	<0.001
Any IVH	12	1.166	1.139	1.193	<0.001	0.0	0.680
Severe IVH	19	1.271	1.207	1.338	<0.001	40.5	0.035
PVL	13	1.110	0.971	1.269	0.128	77.3	<0.001
Severe IVH/PVL	3	1.158	1.023	1.310	0.020	80.8	0.005
Any ROP	4	1.025	0.870	1.207	0.767	66.3	0.031
Severe ROP	20	1.143	1.065	1.226	<0.001	79.2	<0.001
Any NEC	11	1.145	1.036	1.266	0.008	60.1	0.003
NEC stage ≥ II	9	1.122	1.039	1.211	0.003	32.9	0.155
Mortality	20	1.227	1.163	1.294	<0.001	83.7	<0.001

Random effects analysis. Risk ratio (RR) > 1 indicates association of male sex with increased risk of the outcome and RR < 1 indicates association of male sex with decreased risk of the outcome. K: number of studies.

## Data Availability

All data relevant to the study are included in the article or uploaded as supplementary information. Additional data are available upon reasonable request.

## Supplementary Materials

The following are available online at https://www.mdpi.com/article/10.3390/antiox10091490/s1, Table S1: Search strategy; Table S2: Characteristics of the included studies; Table S3: Subgroup analyses based on continent; Table S4: Meta-regression analysis (continuous covariates); Figure S1: Flow diagram of the systematic search; Figure S2: Meta-analysis of the association between male sex of preterm infants and risk of bronchopulmonary dysplasia (BPD), defined as oxygen requirement on postnatal day 28; Figure S3: Meta-analysis of the association between male sex of preterm infants and risk of bronchopulmonary dysplasia (BPD), defined as oxygen requirement at the postmenstrual age of 36 weeks; Figure S4: Meta-analysis of the association between male sex and risk of intraventricular hemorrhage (IVH, grade 1–4) in preterm infants; Figure S5: Meta-analysis of the association between male sex and risk of severe intraventricular hemorrhage (IVH grade 3–4) in preterm infants; Figure S6: Meta-analysis of the association between male sex and risk of periventricular leukomalacia (PVL) in preterm infants; Figure S7: Meta-analysis of the association between male sex of preterm infants and risk of severe intraventricular hemorrhage (IVH grade 3–4) or periventricular leukomalacia (PVL); Figure S8: Meta-analysis of the association between male sex of preterm infants and risk of retinopathy of prematurity (ROP, any stage); Figure S9: Meta-analysis of the association between male sex in preterm infants and risk of severe retinopathy of prematurity (ROP grade ≥ 3 or requiring treatment); Figure S10: Meta-analysis of the association between male sex and risk of necrotizing enterocolitis (NEC, any stage) in preterm infants; Figure S11: Meta-analysis of the association between male sex and risk of necrotizing enterocolitis (NEC, stage ≥ II) in preterm infants; Figure S12: Meta-analysis of the association between male sex and risk of mortality before discharge in preterm infants; Figure S13: Funnel plot for publication bias analysis for the studies included in the different meta-analyses.
