# Supplementary material for: Male Disadvantage in Oxidative Stress-Associated Complications of Prematurity: A Systematic Review, Meta-Analysis and Meta-Regression
Source: Antioxidants (Basel). 2021 Sep 18;10(9):1490. doi: 10.3390/antiox10091490 (PMC8465696; doi:10.3390/antiox10091490)
Supplement: Supplementary file 1 [file antioxidants-10-01490-s001.zip › antioxidants-1380444-supplementary.pdf]

Supplementary Material

Male Disadvantage in Oxidative Stress-Associated Complications of Prematurity: A Systematic Review, Meta-analysis and Meta-regression

Elke van Westering-Kroon, Maurice J Huizing, Eduardo Villamor-Martínez and Eduardo Villamor

Supplementary Table 1. **Search strategy**

**Pubmed**
(Sex[MESH] OR Sex Characteristics[MESH] OR Sex Distribution[MESH] OR "Sex Characteristic*"[tiab] OR "Gender Difference*"[tiab] OR "Sex Dimorphism*"[tiab] OR "Gender Characteristic*"[tiab] OR "Sex Difference*"[tiab] OR "Sex Distribution*"[tiab] OR "Gender Distribution*"[tiab] OR "Male Disadvantage"[tiab] OR "Female Advantage"[tiab])<br>AND<br>(<br>(Premature Infant[MESH] OR Extremely Premature Infant[MESH] OR Premature Birth[MESH] OR Low Birth Weight Infant[MESH] OR Very Low Birth Weight Infant[MESH] OR "Premature Infant*"[tiab] OR "Preterm Infant*"[tiab] OR "Preterm Infant"[tiab] OR "Premature Infants"[tiab] OR "Neonatal Prematurity"[tiab] OR "Low-Birth-Weight Infant*"[tiab] OR "Infants, Low-Birth-Weight"[tiab] OR "Low Birth Weight Infant"[tiab] OR "Low-Birth-Weight Infants"[tiab] OR "Low Birth Weight*"[tiab])<br>OR<br>(Retinopathy of Prematurity[mesh] OR Prematurity Retinopathies[tiab] OR Prematurity Retinopathy[tiab] OR Retrolental Fibroplasia[tiab] OR Retrolental Fibroplasia*[tiab])<br>OR<br>(Necrotizing enterocolitis[mesh] OR Necrotizing enterocolitis[tiab])<br>OR<br>(Periventricular Leukomalacia [mesh] OR Leukomalacia*[tiab] OR Periventricular Encephalomalacia*[tiab] OR Periventricular Leukomalacia*[tiab] OR Neonatal Cerebral Leukomalacia*[tiab] OR Cystic Periventricular Leukomalacia*[tiab])<br>OR<br>(Bronchopulmonary Dysplasia [mesh] OR Bronchopulmonary Dysplasia[tiab] OR "Chronic Lung Disease"[tiab])<br>OR<br>(Cerebral Intraventricular Hemorrhage [mesh] OR Intraventricular Hemorrhage[tiab] OR Intraventricular Haemorrhage[tiab] OR Intracranial Hemorrhage[tiab] OR Intracranial Haemorrhage[tiab])<br>)

Embase
(
(exp Sex Characteristics/ OR exp Sex Distribution / OR exp Sex Factors/)
OR ("male disadvantage" or "female advantage" or gender or sex or "gender difference" or "gender differences" or "sex difference" or "sex differences" or "gender differential" or "gender differentials" or "sex differential" or "sex differentials" or "sexual dimorphism" or "sexually dimorphic")
)
AND
(
(exp Premature infant/ OR exp Premature Birth OR exp Low Birth Weight Infant/ OR Extremely Low Birth Weight Infant/)
OR
(bronchopulmonary dysplasia/ OR enterocolitis, necrotizing/ OR Cerebral Intraventricular Hemorrhage/ OR "Intraventricular Hemorrhage" OR Leukomalacia, Periventricular/ OR "Retinopathy of Prematurity"/)
)

Supplementary Table 2. Characteristics of the included studies

First author	Year	Country	Total n	Male to female ratio	Gestational age (weeks)	Median year cohort	Prospective?	Singleton/Twins	Obstetric and perinatal variables	Postnatal variables and outcomes	NOS Selection	NOS Comparability	NOS Outcome	NOS Total
Bertino[1]	2009	Italy	262	0,87	30.4	1997	No	Both	BW, GA, SGA (P10)	BPD36, LOS, PDA	4	2	3	9
Binet[2]	2012	Canada	2744	1,17	25.7	2003	No	Both	ACS, Outborn	BPD36, IVH 3-4, Mortality, NEC>1, sROP	4	1	3	8
Boghossian[3]	2018	USA	205731	1,12	26.0	2011	No	Both	BW, ACS, Chorio, HDP, Intubation birth, Prenatal care	BPD36, EOS, IVH 3-4, LOS, Mortality, NEC>1/LIP, PDA, Pneumothorax, Postnatal steroids, RDS, Surfact	4	1	3	8
Chen[4]	2018	China	370	1,96	33.1	2011	No	Both	Abruption, ACS, BW, C-section, Fetal distress, GA, HDP, Mat diabetes, PremROM	Mortality, Mec Ventil, RDS, Surfact	4	2	3	9
Derzbach[5]	2005	Hungary	140	0,94	29.0	2003	No	Both	BW, GA	IVH 1-4, Hypotension, NEC, PDA, RDS, UOS	3	2	3	8
Deulofeut [6]	2007	USA	497	0,94	27.0	2001	No	Both	ACS, BW, C-section, GA, Prenatal care, SGA(P10)	IVH 1-4, BPD36, IVH 3-4, Mortality, PVL, ROP (all), sROP	4	2	3	9
Elsmen[7]	2004	Sweden	236	1,23	26.4	1997	No	Both	ACS, BW, C-section,GA, HDP, Intubation birth, SGA(P10), Spont pregnancy	BPD36, Hypotension, PDA,Surfact	4	1	3	8
Gagliardi[8]	2021	Network	20924	1,13	26.9	2011	No	Twins	BW, ACS, C-section, GA, Outborn, SGA (P10)	BPD36, IVH 3-4/PVL, sROP	3	2	3	8
Garfinkle [9]	2018	Canada	59523	0,94	26.0	2012	No	Both	Apgar5<7, C-section, GA, HDP, Mat age, Mat diabetes, Outborn, SGA(P10)	BPD36, IVH 3-4/PVL, LOS, Mortality, NEC>1, sROP	4	2	3	9
Griesmaier[10]	2014	Austria	156	1,20	30.5	2010	No	Both	ACS, BW, GA, SGA(P10)	BPD28,EOS,LOS,NEC, PDA, PremROM,	4	1	3	8
Harris[11]	2019	UK	319	1,03	26.9	1999	No	Both	ACS, BW, GA, Mat age, Smoking	BPD28, BPD36, NEC (all), PDA, Pneumothorax, Postnatal steroids, Pulm Hem	3	1	3	7
Hintz[12]	2006	USA	2553	0,91	25.4	1999	Yes	Both	ACS,BW, Apgar5<5, C-section,GA, Intubation birth, Mat age, Outborn, ProlROM, Resuscit, SGA(P10)	BPD36, EOS, IVH3-4, LOS, NEC>1, PDA, Postnatal steroids, PVL, sROP, Surfact	4	2	3	9
Huang[13]	2012	Taiwan	195	1,50	28.4	2002	No	Both		PVL, sROP	4	1	3	8
Ito[14]	2017	Japan	38023	1,04	28.0	2008	No	Both	ACS, BW, Chorio, Gest diabetes, HDP, Mat age, PremROM	BPD28, BPD36, LIP, IVH 1-4, Mortality, NEC, PDA, PVL, RDS, sROP	4	1	3	8
Jennische[15]	2003	Sweden	64	1,29	28.9	1988	No	Both	BW, GA, SGA(P10), Resuscit	BPD28, PDA, Pneumothorax, RDS, UOS	4	2	3	9
Jones[16]	2007	Canada	3117	1,27	27.4	1997	Yes	Both	ACS, ,Outborn, SGA(P10)	BPD36, EOS, IVH 3-4, LOS, M vent, Mortality, NEC>1, PDA, sROP	4	2	3	9
Kent[17]	2012	Australia	2549	1,21	26.3	2001	No	Both	ACS, Apgar5<7, C-section, Fetal Distress, HDP, Intrapartum hemo, IUGR, Outborn, ProlROM, SGA(P10)	BPD36, IVH 3-4,Mortality, NEC (all), PDA, Pneumothorax, Postnatal steroids, sROP, Surfact	4	2	3	9
Lauterbach[18]	2001	USA	51	0,70	32.3	1990	No	Both	BW, C-section, GA, Gest diabetes, HDP, Mat age	BPD28, Hypotension, PDA, Pneumothorax, Postnatal steroids, UOS, Surfact	3	1	3	7
Lavoie[19]	1989	Canada	22	0,83	27.0	1988	No	Both	BW, C-section, GA	BPD28,BPD36, IVH 1-4, Mec Ventil, Surfact	3	1	3	7
Mansson [20]	2015	Sweden	398	1,20	31.4	2006	No	Both	Mat age, SGA (-2SD)	BPD36, IVH3-4/PVL, NEC (all), sROP	4	2	3	9

First author	Year	Country	Total n	Male to female ratio	Gestational age (weeks)	Median year cohort	Prospective?	Singleton/Twins	Obstetric and perinatal variables	Postnatal variables and outcomes	NOS Selection	NOS Comparability	NOS Outcome	NOS Total
Melamed[21]	2009	Israel	5408	0,97	35.0	2001	No	Twins	BW, C-section, GA, HDP, Mat age, BW, GA, Mat diabetes, Mother>35, SGA(P10)	IVH 1-4, NEC (all), RDS	3	2	3	8
Mohamed[22]	2010	USA	104847	1,03	VLBW	2000	No	Both	HDP, PDA,	IVH 1-4, IVH 3-4, Mortality, NEC (all), Pulm hem, PVL, RDS	4	2	3	9
Neubauer[23]	2012	Austria	408	1,18	31.0	2006	No	Both	ACS, Apgar1<3, Apgar5<3, C-section, ProlROM, Outborn, SGA(P10), Smoking	BPD36, EOS, LOS, NEC>1, PDA, sROP, Surfact	4	1	3	8
Peacock[24]	2012	UK	797	1,16	26.5	2000	No	Both		IVH 3-4, sROP,	3	1	3	7
Ramos Navarro[25]	2020	Spain	389	1,48	28.5	2015	Yes	Both	Intubation birth	BPD36, Mec vent, Mortality, Pneumothorax, Surfact	4	1	3	8
Shim[26]	2017	Korea	2163	1,07	27.2	2014	No	Both	ACS, BW, Chorio, C-section, GA, HDP, Mat diabetes, Outborn, SGA(P10)	BPD36, IVH 3-4, Mortality, NEC>1, PVL, sROP	4	1	3	8
Shinwell[27]	2007	Israel	8858	1,08	28.6	1999	No	Both	ACS, BW, Chorio, C-section, GA, Infertility therapy, Intrapartum hemo, PremROM, prenatal care, Preterm Labor	BPD36, IVH 3-4, Mortality, Pneumothorax, PVL, RDS, sROP	4	2	3	9
Skiold[28]	2014	Sweden	107	1,28	25.5	2006	No	Both		IVH 1-4, IVH 3-4,sROP	4	1	3	8
Spinillo[29]	2009	Italy	754	1,09	30.3	1997	No	Both	Abruption, ACS, BW, Chorio, C-section, Fetal distress, GA, HDP, Mat age, Placenta previa, PremROM, SGA(P10), Smoking	IVH 1-4, IVH 3-4, PVL	4	2	3	9
Stark[30]	2009	Australia	43	1.15	29	2007	Yes	Single	ACS, Chorio, C-section, HDP, SGA(P10)		4	1	3	8
Stark[31]	2011	Australia	60	1,07	29	2009	Yes	Both	SGA (P10)	IVH 3-4, Mortality, PDA	4	1	3	8
Steen[32]	2013	Sweden	2419	1,18	30	2002	No	Twins	HDP,	BPD36, IVH 3-4, Pneumothorax, RDS, UOS	3	1	3	7
Stevenson[33]	2000	USA	6738	0,99	28.5	1992	No	Both	ACS, Adol mother, Ant AB, Intrapartum hemo, Apgar1<3, Apgar5<3, C-section, Intubation birth, Mother Married, Mother>35, PremROM, prenatal care, ProlROM, Resuscit	IVH 1-4, BPD28,BPD36, Mec Ventil, Meningitis, Mortality, NEC>1, PDA, PVL, RDS, UOS, IVH 3-4, Surfact, Urine Infect	4	1	3	8
Stimac [34]	2019	Croatia	159	1,12	30	2013	No	Both	C-section, HDP, Mat diabetes	IVH 1-4, RDS	4	0	3	7
Tioseco[35]	2006	USA	874	1,00	30.9	1998	No	Both		BPD36, IVH 1-4, IVH 3-4, Mortality, NEC (all), PDA, PVL, ROP (all), UOS	4	2	3	9
Tottman[36]	2020	New Zealand	478	1,22	28	2007	Yes	Both	ACS , Outborn, ProlROM,	BPD36, Mortality, IVH 3-4, NEC>1, UOS, sROP	4	1	3	8
Walker[37]	2012	Canada	262	1,15	28.6	2005	No	Single	C-section, HDP, SGA(P10), SGA(P3)		4	1	3	8
Wang[38]	2018	Taiwan	3507	NA	<32	2011	No	Both		BPD36, PVL, sROP	4	1	3	8
Zhao[39]	2017	USA	114960	1,00	<37	2000	No	Twins	Apgar5<7	Mortality, Mec Ventil, RDS	3	2	3	8
Zisk[40]	2011	USA	12807	1,10	29.4	2005	No	Both	Apgar5<3, SGA(p10)	BPD36, IVH 3-4, Mortality, NEC (all), PDA Pneumothorax, Postnatal steroids, sROP, Surfact, UOS	4	2	3	9

First author	Year	Country	Total n	Male to female ratio	Gestational age (weeks)	Median year cohort	Prospective?	Singleton/Twins	Obstetric and perinatal variables	Postnatal variables and outcomes	NOS Selection	NOS Comparability	NOS Outcome	NOS Total
Zozaya[41]	2019	Spain	21768	1,10	28.6	2010	No	Both	ACS, C-section, SGA(-2SD)	BPD36, LOS, Mortality, NEC>1, PDA, ROP (all), PVL	4	1	3	8

ACS: antenatal corticosteroids; Adol: adolescent; Ant: antenatal; BPD28: BPD defined as oxygen requirement on postnatal day 28; BPD36: defined as oxygen requirement at the postmenstrual age of 36 weeks; BW: birth weight; Chorio: chorioamnionitis; EOS: early onset sepsis; Gest: gestational; HDP: hypertensive disorders of pregnancy; Hemo: hemorrhage; Infect: infection; IUGR: intrauterine growth restriction; IVH: intraventricular hemorrhage; LIP: local intestinal perforation; LOS: late onset sepesis; Mat: maternal; Mec Ventil: mechanical ventilation; NEC: necrotizing enterocolitis; NOS: Newcastle Ottawa Scale; P3: 3rd percentile; P10: 10th percentile; PDA: patent ductus arteriosus; PremROM: premature rupture of membranes; ProlROM: prolonged rupture of membranes; Pulm hem: pulmonary hemorrhage; PVL: periventricular leukomalacia; RDS: respiratory distress syndrome; Resuscit: resuscitation at birth; ROP: retinopathy of prematurity; SD: standard deviation; SGA: small for gestational age; Single: singleton; Spont: spontaneous; sROP: severe ROP; Surfact: surfactant; UOS: undefined onset sepsis.

Supplementary Table 3. Subgroup analyses based on continent

Meta-analysis	Criteria for subgrouping	K	RR	95% CI		P	Heterogeneity		Meta-regression	
				Lower limit	Upper limit		I^2 (%)	P	P-value	R^2 analog
BPD36	America	11	1.183	1.143	1.224	<0.001	45.7	0.048	0.145	0.0
	Asia	4	1.305	1.159	1.469	<0.001	82.7	0.001		
	Europe	8	1.346	1.203	1.506	<0.001	25.4	0.226		
	Oceania	2	1.066	0.950	1.197	0.274	0.0	0.486		
Any IVH	America	6	1.173	1.142	1.205	<0.001	0.0	0.701	0.516	0.0
	Asia	2	1.150	1.069	1.237	<0.001	6.1	0.302		
	Europe	4	1.081	0.898	1.301	0.410	0.0	0.395		
Severe IVH	America	10	1.248	1.169	1.333	<0.001	60.5	0.007	0.747	0.0
	Asia	2	1.342	1.212	1.485	<0.001	0.0	0.469		
	Europe	4	1.391	1.105	1.752	0.005	0.0	0.427		
	Oceania	3	1.105	0.621	1.965	0.735	40.9	0.184		
PVL	America	6	0.939	0.796	1.108	0.456	48.1	0.086	0.048	0.5
	Asia	5	1.208	1.032	1.414	0.018	58.4	0.047		
	Europe	2	1.303	0.980	1.731	0.068	16.8	0.273		
Severe ROP	America	8	1.121	1.038	1.212	0.004	68.4	0.002	0.827	0.0
	Asia	5	1.229	0.936	1.612	0.137	93.5	<0.001		
	Europe	5	1.096	0.908	1.324	0.338	15.2	0.318		
	Oceania	2	1.252	0.993	1.580	0.058	0.0	0.350		
Any NEC	America	4	1.127	0.994	1.279	0.062	77.1	0.004	0.256	0.17
	Asia	2	1.289	0.974	1.706	0.076	70.3	0.066		
	Europe	4	0.933	0.653	1.332	0.703	0.0	0.0		
	Oceania	1	1.003	0.768	1.312	0.980	0.0	1.000		
Mortality	America	11	1.242	1.152	1.338	<0.001	90.8	<0.001	0.947	0.0
	Asia	4	1.200	1.087	1.325	<0.001	50.9	0.106		
	Europe	2	1.228	1.152	1.308	<0.001	0.0	0.443		
	Oceania	3	1.238	1.069	1.434	0.004	0.0	0.679		

BPD36: bronchopulmonary dysplasia defined as oxygen requirement at the postmenstrual age of 36 weeks; GA: gestational age; IVH: intraventricular hemorrhage; K: number of studies; NEC: necrotizing entrocolitis; PVL: periventricular leukomalacia; ROP: retinopathy of prematurity.

Supplementary Table 4. Meta-regression analysis (continuous covariates)

Meta-analysis	Covariate	K	Coefficient	Standard Error	P	R^2 analog
BPD36	GA cohort (weeks)	25	0.025	0.013	0.065	0.05
	Median year of cohort	26	-0.005	0.003	0.076	0.01
Any IVH	GA cohort (weeks)	11	0.033	0.019	0.079	0.0
	Median year of cohort	12	-0.002	0.003	0.531	0.0
Severe IVH	GA cohort (weeks)	18	-0.014	0.024	0.567	0.0
	Median year of cohort	19	0.002	0.005	0.725	0.0
PVL	GA cohort (weeks)	11	0.038	0.063	0.546	0.0
	Median year of cohort	13	0.009	0.010	0.380	0.0
Severe ROP	GA cohort (weeks)	19	0.005	0.017	0.761	0.0
	Median year of cohort	20	0.001	0.007	0.901	0.0
Any NEC	GA cohort (weeks)	10	-0.012	0.023	0.594	0.0
	Median year of cohort	11	0.029	0.015	0.052	0.33
Mortality	GA cohort (weeks)	18	0.051	0.014	<0.001	0.75
	Median year of cohort	20	-0.009	0.004	0.019	0.37

Random effects (method of moments), univariate meta-regression. Coefficient indicates the change in the log of the RR of the association between male sex and the corresponding outcome for a unit change in the predictor covariate. R^2 analog: total between-study variance explained by the moderator. BPD36: bronchopulmonary dysplasia defined as oxygen requirement at the postmenstrual age of 36 weeks; GA: gestational age; IVH: intraventricular hemorrhage; K: number of studies; NEC: necrotizing entrocolitis; ROP: retinopathy of prematurity.

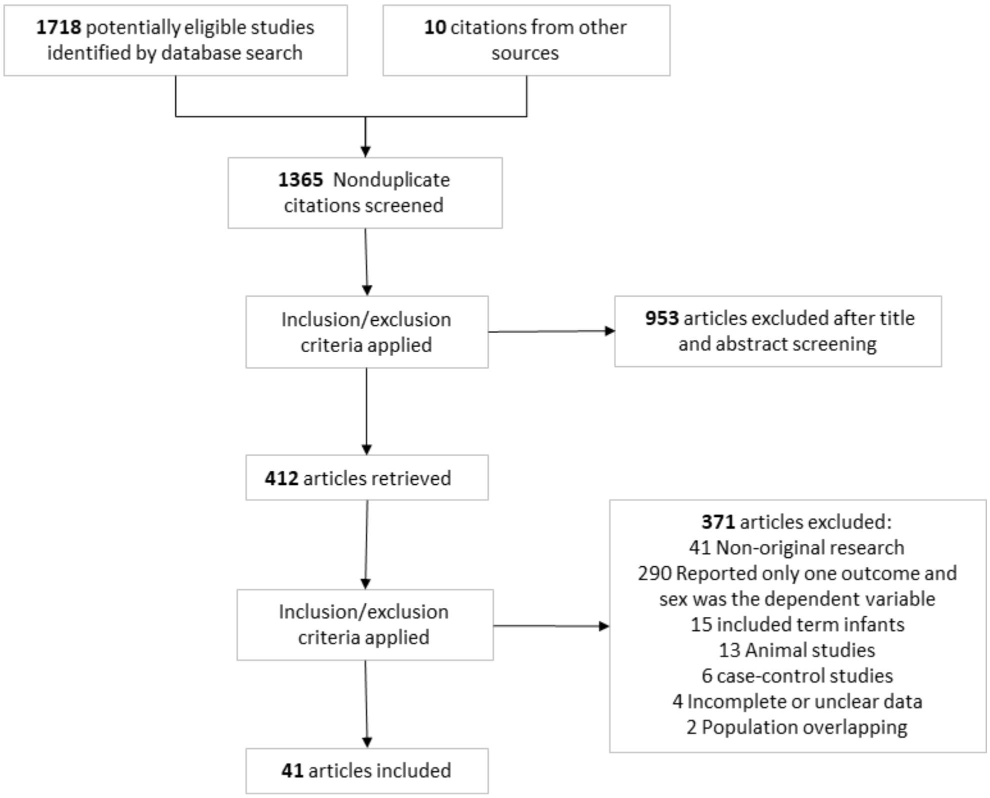

Supplementary Figure 1. Flow diagram of the systematic search.

Any BPD

Study name	Year	Statistics for each study			
		Risk ratio	Lower limit	Upper limit	p-Value
Lauterbach	2001	0.282	0.014	5.586	0.406
Jennische	2003	0.389	0.150	1.009	0.052
Lavoie	1998	0.750	0.359	1.568	0.445
Harris	2019	1.165	1.049	1.293	0.004
Ito	2017	1.181	1.148	1.216	0.000
Griesmaier	2014	1.253	0.215	7.292	0.802
Stevenson	2000	1.330	1.241	1.425	0.000
**Overall**		**1.200**	**1.091**	**1.319**	**0.000**

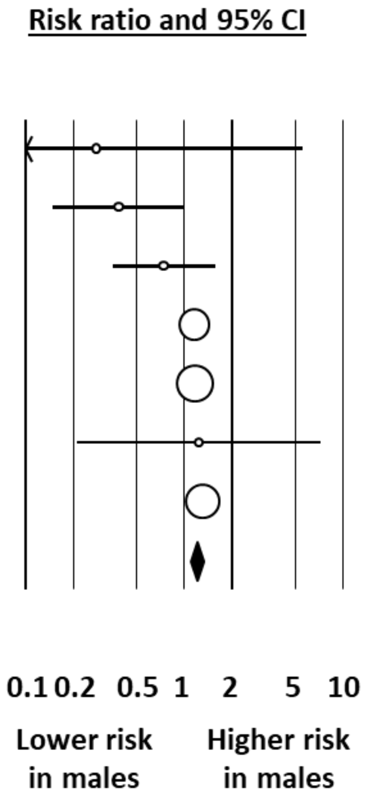

Supplementary Figure 2. Meta-analysis of the association between male sex of preterm infants and risk of bronchopulmonary dysplasia (BPD), defined as oxygen requirement on postnatal day 28.

CI: confidence interval.

Moderate/severe BPD

Study name	Year	Risk ratio	Lower limit	Upper limit	p-Value
Ramos Navarro	2020	0.798	0.610	1.045	0.100
Lavoie	1998	0.800	0.310	2.062	0.644
Mansson	2015	0.953	0.662	1.372	0.797
Kent	2012	1.054	0.934	1.188	0.394
Tioseco	2006	1.095	0.761	1.575	0.626
Garfinkle	2020	1.111	1.063	1.162	0.000
Shim	2017	1.131	1.014	1.261	0.027
Jones	2005	1.155	1.015	1.314	0.028
Deulofeut period I	2007	1.166	0.936	1.454	0.171
Binet	2011	1.175	1.077	1.281	0.000
Gagliardi	2021	1.185	1.135	1.238	0.000
Boghossian	2018	1.187	1.172	1.203	0.000
Zisk	2011	1.190	1.110	1.276	0.000
Hintz	2006	1.196	1.109	1.289	0.000
Tottman	2020	1.226	0.814	1.847	0.329
Ito	2017	1.232	1.176	1.289	0.000
Harris	2019	1.248	1.029	1.513	0.024
Stevenson	2000	1.363	1.220	1.523	0.000
Wang	2018	1.390	1.200	1.610	0.000
Zozaya	2019	1.396	1.303	1.495	0.000
Steen	2013	1.470	1.081	1.999	0.014
Shinwell	2007	1.539	1.367	1.733	0.000
Neubauer	2012	1.598	0.934	2.735	0.087
Deulofeut period II	2007	1.696	1.143	2.519	0.009
Bertino	2009	1.721	0.803	3.690	0.163
Elsmen	2004	1.916	1.214	3.023	0.005
**Overall**		**1.219**	**1.176**	**1.264**	**0.000**

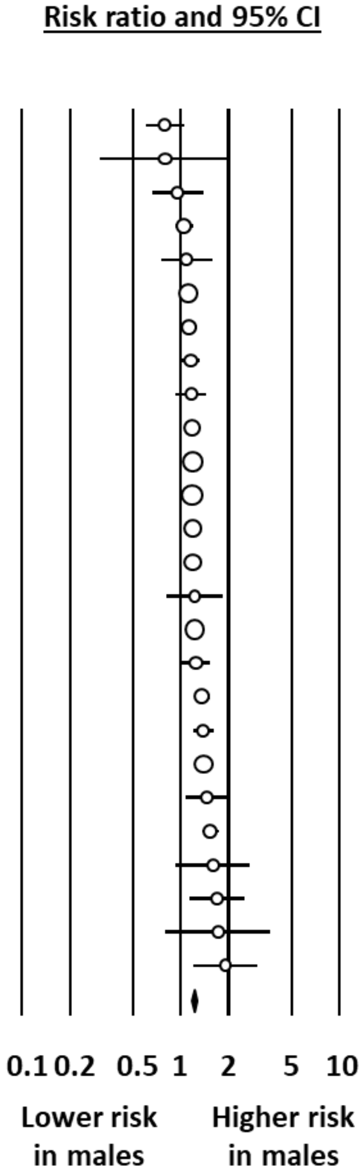

Supplementary Figure 3. Meta-analysis of the association between male sex of preterm infants and risk of bronchopulmonary dysplasia (BPD), defined as oxygen requirement at the postmenstrual age of 36 weeks.

CI: confidence interval.

Any IVH

Study name	Year	Risk ratio	Lower limit	Upper limit	p-Value
Skiold	2014	0.783	0.516	1.189	0.252
Spinillo	2009	1.111	0.789	1.563	0.547
Deulofeut period II	2007	1.127	0.809	1.572	0.479
Ito	2017	1.140	1.082	1.200	0.000
Stevenson	2000	1.157	1.083	1.236	0.000
Mohamed	2010	1.174	1.140	1.209	0.000
Derzbach	2005	1.197	0.761	1.882	0.436
Lavoie	1998	1.200	0.204	7.054	0.840
Stimac	2019	1.208	0.881	1.656	0.240
Deulofeut period I	2007	1.221	0.841	1.772	0.293
Melamed	2009	1.334	0.993	1.791	0.055
Tioseco	2006	1.687	1.095	2.598	0.018
**Overall**		**1.166**	**1.139**	**1.193**	**0.000**

(Statistics for each study)

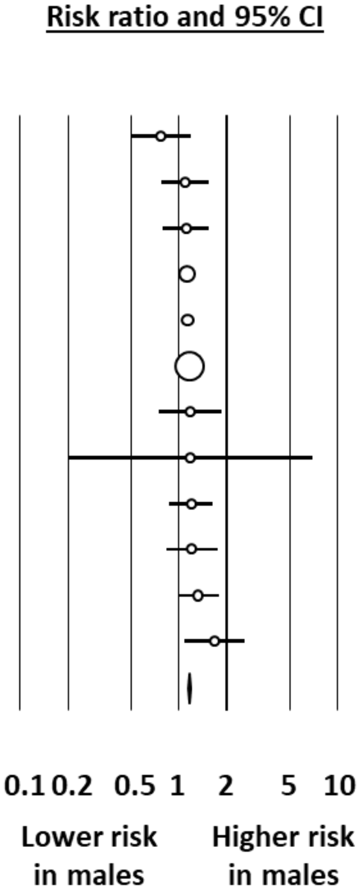

Supplementary Figure 4. Meta-analysis of the association between male sex and risk of intraventricular hemorrhage (IVH, grade 1-4) in preterm infants.

CI: confidence interval.

Severe IVH

Study name	Year	Risk ratio	Lower limit	Upper limit	p-Value
Tottman	2020	0.613	0.263	1.428	0.257
Zisk	2011	1.077	0.875	1.327	0.483
Stevenson	2000	1.085	0.956	1.231	0.207
Binet	2011	1.096	0.951	1.263	0.207
Spinillo	2009	1.142	0.542	2.407	0.727
Steen	2013	1.148	0.798	1.653	0.457
Shim	2017	1.254	1.017	1.546	0.035
Hintz	2006	1.261	1.059	1.500	0.009
Jones	2005	1.281	1.013	1.621	0.039
Kent	2012	1.303	1.050	1.617	0.016
Boghossian	2018	1.311	1.274	1.349	0.000
Mohamed	2010	1.359	1.290	1.431	0.000
Shinwell	2007	1.370	1.220	1.539	0.000
Skiold	2014	1.371	0.426	4.406	0.596
Deulofeut period II	2007	1.698	0.739	3.900	0.212
Peacock	2012	1.712	1.221	2.400	0.002
Deulofeut period I	2007	1.798	1.028	3.143	0.040
Tioseco	2006	2.024	0.959	4.272	0.064
Stark 2011	2011	2.806	0.309	25.476	0.359
**Overall**		**1.271**	**1.207**	**1.338**	**0.000**

Statistics for each study

Risk ratio and 95% CI

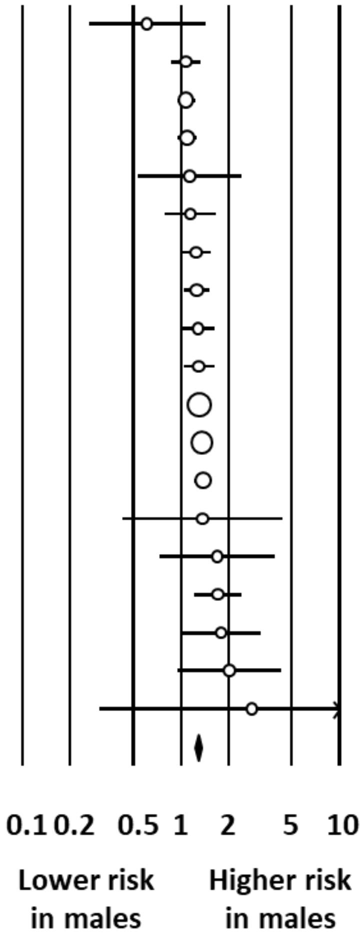

Supplementary Figure 5. Meta-analysis of the association between male sex and risk of severe intraventricular hemorrhage (IVH grade 3-4) in preterm infants.

CI: confidence interval.

PVL

Study name	Year	Statistics for each study			
		Risk ratio	Lower limit	Upper limit	p-Value
Deulofeut period I	2007	0.266	0.092	0.771	0.015
Deulofeut period II	2007	0.653	0.222	1.921	0.439
Mohamed	2010	0.892	0.841	0.946	0.000
Stevenson	2000	0.999	0.840	1.189	0.993
Tioseco	2006	1.012	0.255	4.020	0.986
Ito	2017	1.029	0.924	1.147	0.599
Huang	2012	1.167	0.610	2.232	0.641
Hintz	2006	1.176	0.861	1.606	0.308
Zozaya	2019	1.235	1.046	1.457	0.013
Wang	2019	1.260	0.810	1.960	0.305
Shinwell	2007	1.324	1.144	1.533	0.000
Shim	2017	1.365	1.068	1.746	0.013
Spinillo	2009	1.929	0.884	4.208	0.099
**Overall**		**1.110**	**0.971**	**1.269**	**0.128**

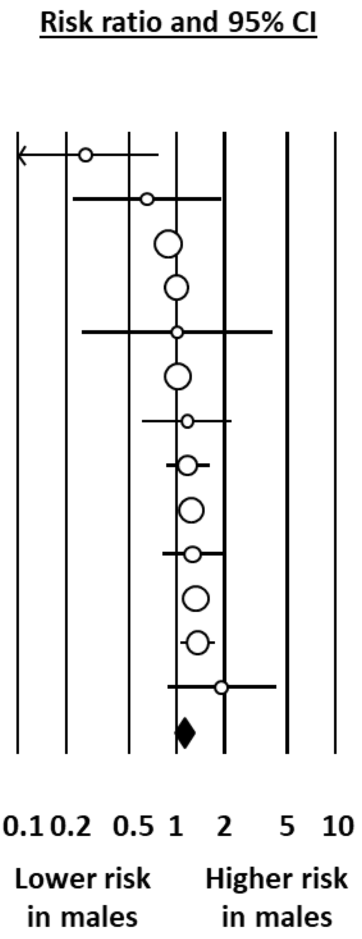

Supplementary Figure 6. Meta-analysis of the association between male sex and risk of periventricular leukomalacia (PVL) in preterm infants.

CI: confidence interval.

Severe IVH/PVL

Study name	Year	Risk ratio	Lower limit	Upper limit	p-Value
Gagliardi	2021	1.072	0.996	1.155	0.064
Garfinkle	2020	1.231	1.183	1.280	0.000
Mansson	2015	1.231	0.734	2.066	0.431
		**1.158**	**1.023**	**1.310**	**0.020**

(Statistics for each study)

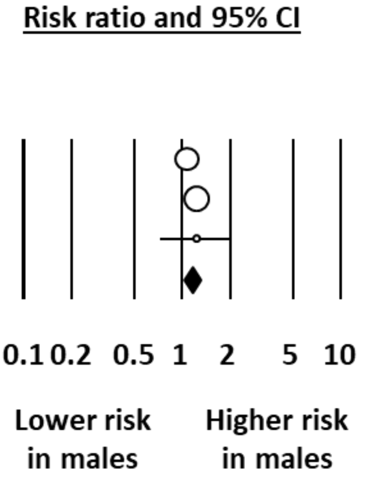

Supplementary Figure 7. Meta-analysis of the association between male sex of preterm infants and risk of severe intraventricular hemorrhage (IVH grade 3-4) or periventricular leukomalacia (PVL).

CI: confidence interval.

Any ROP

Study name	Year	Risk ratio	Lower limit	Upper limit	p-Value
Tioseco	2006	0.817	0.657	1.017	0.070
Zozaya	2019	0.995	0.943	1.049	0.842
Deulofeut period I	2007	1.076	0.863	1.341	0.515
Deulofeut period II	2007	1.519	1.060	2.176	0.023
		1.025	0.870	1.207	0.767

(Statistics for each study)

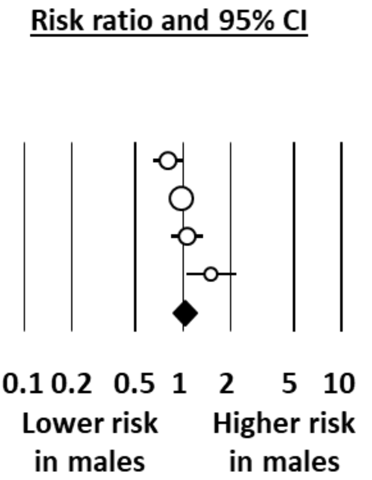

Supplementary Figure 8. Meta-analysis of the association between male sex of preterm infants and risk of retinopathy of prematurity (ROP, any stage).

CI: confidence interval.

Severe ROP

Study name	Year	Statistics for each study			
		Risk ratio	Lower limit	Upper limit	p-Value
Neubauer	2012	0.508	0.188	1.371	0.181
Shim	2017	0.886	0.728	1.078	0.227
Peacock	2012	0.925	0.619	1.383	0.705
Deulofeut period I	2007	0.941	0.468	1.895	0.866
Jones	2005	1.008	0.823	1.234	0.940
Shinwell	2007	1.025	0.882	1.191	0.747
Ito	2017	1.035	0.986	1.087	0.166
Boghossian	2018	1.043	1.010	1.077	0.010
Garfinkle	2020	1.067	0.967	1.177	0.198
Gagliardi	2021	1.090	0.992	1.197	0.072
Mansson	2015	1.107	0.839	1.462	0.472
Binet	2011	1.196	1.008	1.418	0.040
Zisk	2011	1.200	1.124	1.282	0.000
Kent	2012	1.215	0.954	1.546	0.114
Hintz	2006	1.249	1.038	1.503	0.018
Huang	2012	1.385	0.957	2.004	0.085
Skiold	2014	1.828	0.761	4.393	0.178
Tottman	2020	1.869	0.783	4.459	0.159
Wang	2020	2.270	1.860	2.770	0.000
Deulofeut period II	2007	2.500	1.022	6.116	0.045
**Overall**		**1.143**	**1.065**	**1.226**	**0.000**

Risk ratio and 95% CI

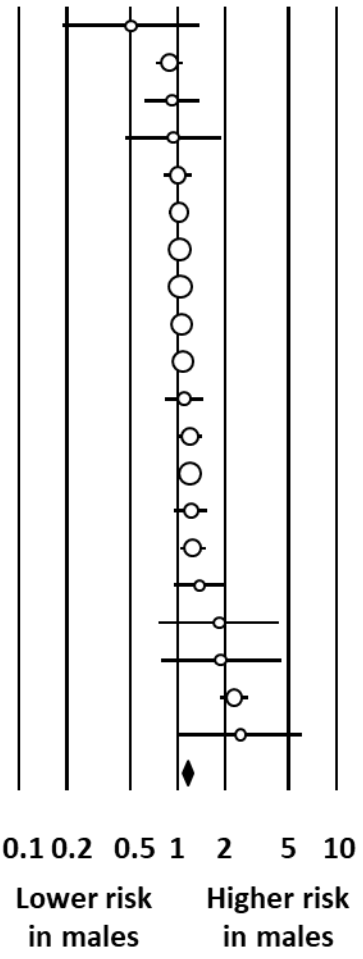

Supplementary Figure 9. Meta-analysis of the association between male sex in preterm infants and risk of severe retinopathy of prematurity (ROP grade≥3 or requiring treatment).

CI: confidence interval.

Any NEC

Study name	Year	Statistics for each study			
		Risk ratio	Lower limit	Upper limit	p-Value
Lauterbach	2001	0.282	0.014	5.586	0.406
Mansson	2015	0.758	0.330	1.745	0.515
Derzbach	2005	0.932	0.584	1.487	0.767
Kent	2012	1.003	0.768	1.312	0.980
Harris	2019	1.049	0.494	2.228	0.900
Mohamed	2010	1.050	0.990	1.114	0.104
Melamed	2009	1.094	0.841	1.423	0.501
Tioseco	2006	1.205	0.685	2.118	0.517
Zisk	2011	1.207	1.147	1.270	0.000
Ito	2017	1.460	1.243	1.715	0.000
Griesmaier	2014	2.512	0.104	60.715	0.571
**Overall**		**1.145**	**1.036**	**1.266**	**0.008**

Risk ratio and 95% CI

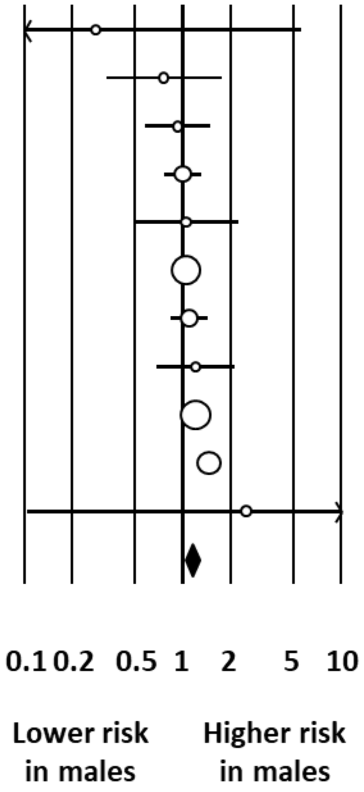

Supplementary Figure 10. Meta-analysis of the association between male sex and risk of necrotizing enterocolitis (NEC, any stage) in preterm infants.

CI: confidence interval.

NEC stage≥II

Study name	Year	Statistics for each study			
		Risk ratio	Lower limit	Upper limit	p-Value
Binet	2011	0.913	0.717	1.163	0.460
Jones	2005	0.930	0.715	1.210	0.590
Zozaya	2019	1.114	1.022	1.214	0.014
Garfinkle	2020	1.125	1.046	1.210	0.002
Stevenson	2000	1.199	0.982	1.462	0.074
Hintz	2006	1.240	0.961	1.600	0.099
Shim	2017	1.253	0.952	1.649	0.108
Neubauer	2012	1.538	0.757	3.128	0.234
Tottman	2020	4.360	1.287	14.766	0.018
**Overall**		**1.122**	**1.039**	**1.211**	**0.003**

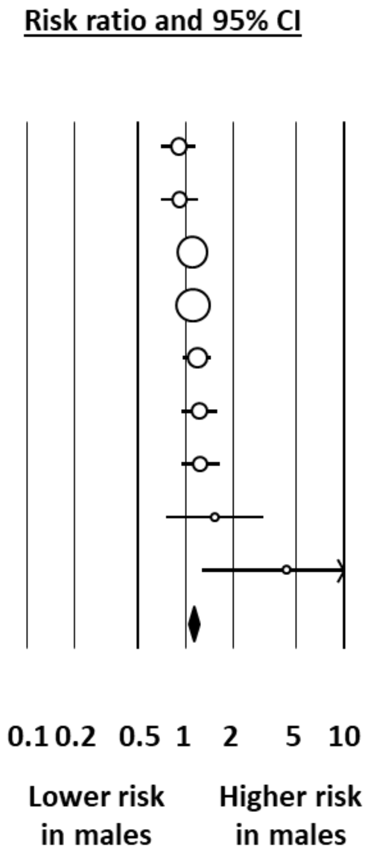

Supplementary Figure 11. Meta-analysis of the association between male sex and risk of necrotizing enterocolitis (NEC, stage ≥II) in preterm infants.

CI: confidence interval.

Mortality

Study name	Year	Risk ratio	Lower limit	Upper limit	p-Value
Stark	2011	0.935	0.302	2.900	0.908
Shim	2017	1.014	0.835	1.231	0.888
Ramos Navarro	2020	1.063	0.733	1.543	0.746
Binet	2011	1.109	0.949	1.295	0.193
Zhao	2017	1.109	1.051	1.170	0.000
Jones	2005	1.133	0.949	1.352	0.167
Garfinkle	2020	1.134	1.051	1.224	0.001
Boghossian	2018	1.150	1.129	1.172	0.000
Ito	2017	1.187	1.099	1.282	0.000
Kent	2012	1.228	1.054	1.430	0.008
Zozaya	2019	1.233	1.156	1.315	0.000
Shinwell	2007	1.301	1.188	1.426	0.000
Tioseco	2006	1.339	0.828	2.167	0.234
Mohamed	2010	1.340	1.300	1.381	0.000
Deulofeut period I	2007	1.380	0.689	2.767	0.364
Stevenson	2000	1.467	1.324	1.625	0.000
Chen	2018	1.474	0.713	3.049	0.295
Zisk	2011	1.510	1.310	1.741	0.000
Tottman	2020	1.572	0.824	2.998	0.170
Deulofeut period II	2007	1.734	0.932	3.227	0.082
**Overall**		**1.227**	**1.163**	**1.294**	**0.000**

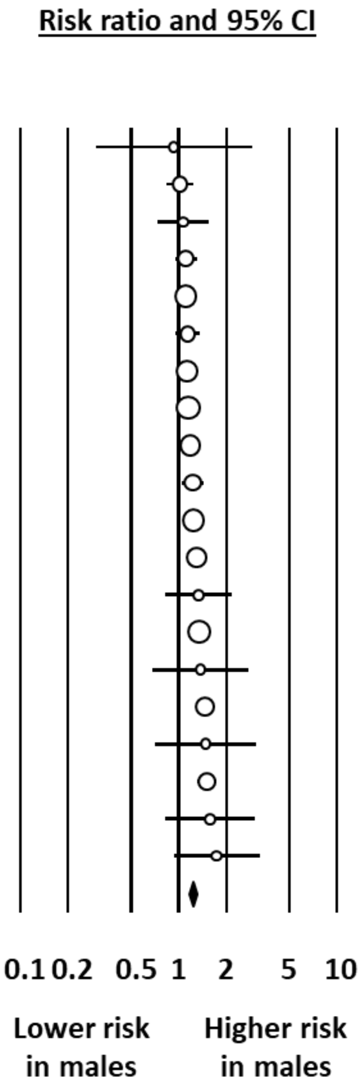

Supplementary Figure 12. Meta-analysis of the association between male sex and risk of mortality before discharge in preterm infants.

CI: confidence interval.

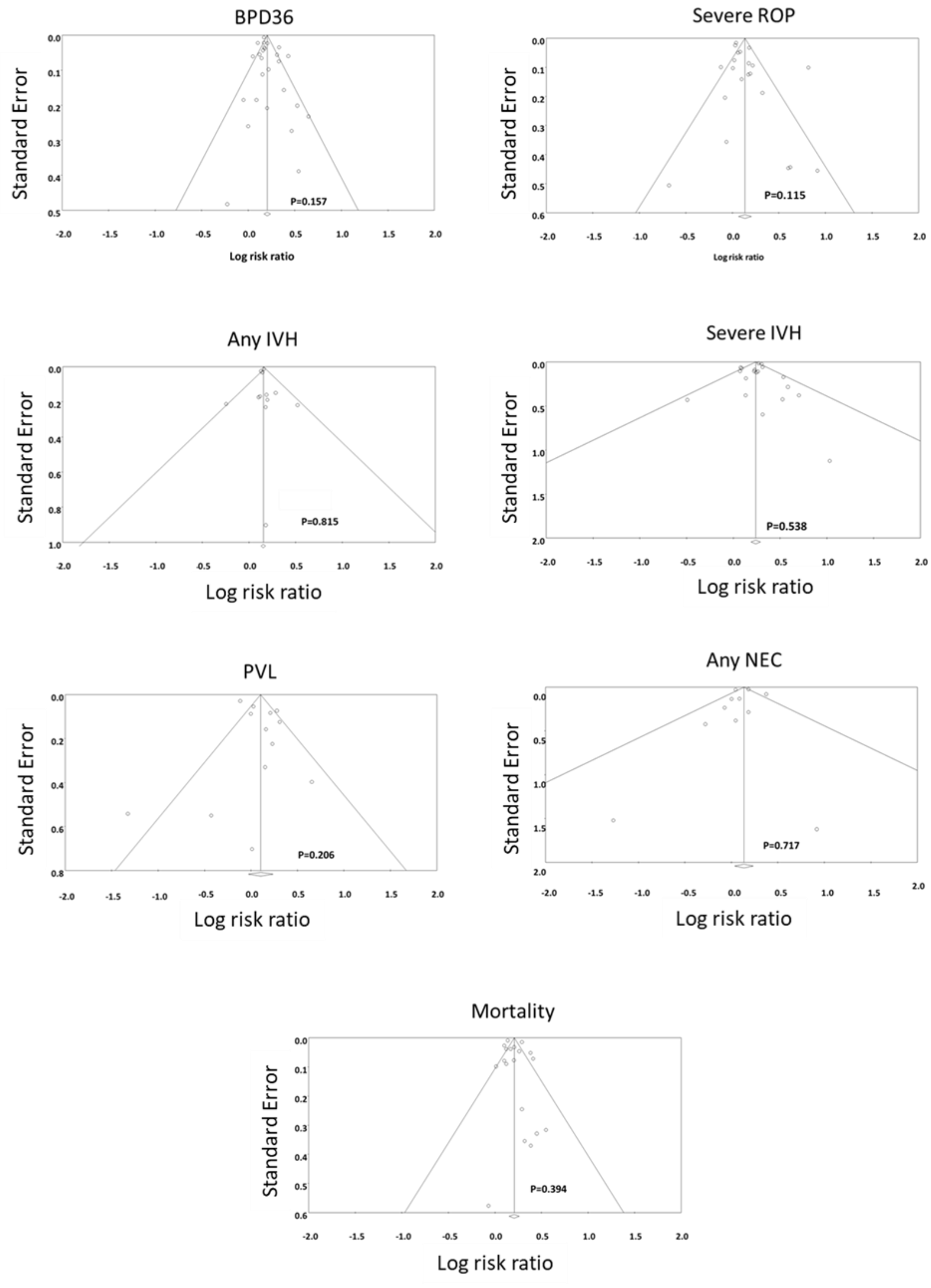

Supplementary Figure 13. Funnel plot for publication bias analysis for the studies included in the different meta-analyses.

BPD36: bronchopulmonary dysplasia defined as oxygen requirement at the postmenstrual age of 36 weeks; ROP; retinopathy of prematurity; IVH: intraventricular hemorrhage; PVL: periventricular leukomalacia; NEC: necrotizing enterocolitis. P-value represents Egger's regression test for funnel plot asymmetry.

List of included studies

1. Bertino E, Coscia A, Boni L, et al. Weight growth velocity of very low birth weight infants: role of gender, gestational age and major morbidities. *Early human development.* 2009;85(6):339-347.
2. Binet M-E, Bujold E, Lefebvre F, Tremblay Y, Piedboeuf B, Network CN. Role of gender in morbidity and mortality of extremely premature neonates. *American journal of perinatology.* 2012;29(03):159-166.
3. Boghossian NS, Geraci M, Edwards EM, Horbar JD. Sex differences in mortality and morbidity of infants born at less than 30 weeks' gestation. *Pediatrics.* 2018;142(6).
4. Chen C, Tian T, Liu L, Zhang J, Fu H. Gender-related efficacy of pulmonary surfactant in infants with respiratory distress syndrome: A STROBE compliant study. *Medicine.* 2018;97(17).
5. Derzbach L, Treszl A, Balogh Á, Vásárhelyi B, Tulassay T. Gender dependent association between perinatal morbidity and estrogen receptor-alpha Pvull polymorphism. *Journal of perinatal medicine.* 2005;33(5):461-462.
6. Deulofeut R, Dudell G, Sola A. Treatment-by-gender effect when aiming to avoid hyperoxia in preterm infants in the NICU. *Acta Paediatrica.* 2007;96(7):990-994.
7. Elsmén E, Pupp IH, Hellström-Westas L. Preterm male infants need more initial respiratory and circulatory support than female infants. *Acta Paediatrica.* 2004;93(4):529-533.
8. Gagliardi L, Rusconi F, Reichman B, et al. Neonatal outcomes of extremely preterm twins by sex pairing: an international cohort study. *Archives of Disease in Childhood-Fetal and Neonatal Edition.* 2021;106(1):17-24.
9. Garfinkle J, Yoon EW, Alvaro R, et al. Trends in sex-specific differences in outcomes in extreme preterms: progress or natural barriers? *Archives of Disease in Childhood-Fetal and Neonatal Edition.* 2020;105(2):158-163.
10. Griesmaier E, Santuari E, Edlinger M, Neubauer V, Waltner-Romen M, Kiechl-Kohlendorfer U. Differences in the maturation of amplitude-integrated EEG signals in male and female preterm infants. *Neonatology.* 2014;105(3):175-181.
11. Harris C, Zivanovic S, Lunt A, et al. Lung function and respiratory outcomes in teenage boys and girls born very prematurely. *Pediatric pulmonology.* 2020;55(3):682-689.
12. Hintz SR, Kendrick DE, Vohr BR, Poole WK, Higgins RD, Network NNR. Gender differences in neurodevelopmental outcomes among extremely preterm, extremely-low-birthweight infants. *Acta paediatrica.* 2006;95(10):1239-1248.
13. Huang H-M, Lin S-A, Chang Y-C, Kuo H-K. Correlation between periventricular leukomalacia and retinopathy of prematurity. *European journal of ophthalmology.* 2012;22(6):980-984.
14. Ito M, Tamura M, Namba F, Japan NRNo. Role of sex in morbidity and mortality of very premature neonates. *Pediatrics International.* 2017;59(8):898-905.
15. Jennische M, Sedin G. Gender differences in outcome after neonatal intensive care: speech and language skills are less influenced in boys than in girls at 6.5 years. *Acta Paediatrica.* 2003;92(3):364-378.
16. Jones HP, Karuri S, Cronin CM, et al. Actuarial survival of a large Canadian cohort of preterm infants. *BMC pediatrics.* 2005;5(1):1-13.
17. Kent AL, Wright IM, Abdel-Latif ME. Mortality and adverse neurologic outcomes are greater in preterm male infants. *Pediatrics.* 2012;129(1):124-131.
18. Lauterbach MD, Raz S, Sander CJ. Neonatal hypoxic risk in preterm birth infants: the influence of sex and severity of respiratory distress on cognitive recovery. *Neuropsychology.* 2001;15(3):411.
19. Lavoie ME, Robaey P, Stauder JE, Glorieux J, Lefebvre F. Extreme prematurity in healthy 5-year-old children: A re-analysis of sex effects on event-related brain activity. *Psychophysiology.* 1998;35(6):679-689.

20. Månsson J, Fellman V, Stjernqvist K, Group ES. Extremely preterm birth affects boys more and socio-economic and neonatal variables pose sex-specific risks. *Acta paediatrica.* 2015;104(5):514-521.
21. Melamed N, Yogev Y, Glezerman M. Effect of fetal sex on pregnancy outcome in twin pregnancies. *Obstetrics & Gynecology.* 2009;114(5):1085-1092.
22. Mohamed MA, Aly H. Male gender is associated with intraventricular hemorrhage. *Pediatrics.* 2010;125(2):e333-e339.
23. Neubauer V, Griesmaier E, Ralser E, Kiechl-Kohlendorfer U. The effect of sex on outcome of preterm infants–a population-based survey. *Acta paediatrica.* 2012;101(9):906-911.
24. Peacock JL, Marston L, Marlow N, Calvert SA, Greenough A. Neonatal and infant outcome in boys and girls born very prematurely. *Pediatric research.* 2012;71(3):305-310.
25. Ramos-Navarro C, Sánchez-Luna M, Zeballos-Sarrato S, Pescador-Chamorro I. Antenatal corticosteroids and the influence of sex on morbidity and mortality of preterm infants. *The Journal of Maternal-Fetal & Neonatal Medicine.* 2020:1-8.
26. Shim S-Y, Cho SJ, Kong KA, Park EA. Gestational age-specific sex difference in mortality and morbidities of preterm infants: a nationwide study. *Scientific reports.* 2017;7(1):1-8.
27. Shinwell ES, Reichman B, Lerner-Geva L, Boyko V, Blickstein I. "Masculinizing" effect on respiratory morbidity in girls from unlike-sex preterm twins: a possible transchorionic paracrine effect. *Pediatrics.* 2007;120(3):e447-e453.
28. Skiöld B, Alexandrou G, Padilla N, Blennow M, Vollmer B, Ådén U. Sex differences in outcome and associations with neonatal brain morphology in extremely preterm children. *The Journal of pediatrics.* 2014;164(5):1012-1018.
29. Spinillo A, Montanari L, Gardella B, Roccio M, Stronati M, Fazzi E. Infant sex, obstetric risk factors, and 2-year neurodevelopmental outcome among preterm infants. *Developmental Medicine & Child Neurology.* 2009;51(7):518-525.
30. Stark MJ, Wright IM, Clifton VL. Sex-specific alterations in placental 11β-hydroxysteroid dehydrogenase 2 activity and early postnatal clinical course following antenatal betamethasone. *American Journal of Physiology-Regulatory, Integrative and Comparative Physiology.* 2009;297(2):R510-R514.
31. Stark MJ, Hodyl NA, Wright IM, Clifton V. The influence of sex and antenatal betamethasone exposure on vasoconstrictors and the preterm microvasculature. *The Journal of Maternal-Fetal & Neonatal Medicine.* 2011;24(10):1215-1220.
32. Steen EE, Källén K, Maršál K, Norman M, Hellström-Westas L. Impact of sex on perinatal mortality and morbidity in twins. *Journal of perinatal medicine.* 2014;42(2):225-231.
33. Stevenson DK, Verter J, Fanaroff AA, et al. Sex differences in outcomes of very low birthweight infants: the newborn male disadvantage. *Archives of disease in childhood-fetal and neonatal edition.* 2000;83(3):F182-F185.
34. Štimac T, Šopić-Rahelić A-M, Ivandić J, Ekinja E, Blickstein I. Effect of gender on growth-restricted fetuses born preterm. *Journal of perinatal medicine.* 2019;47(6):677-679.
35. Tioseco JA, Aly H, Essers J, Patel K, El-Mohandes AA. Male sex and intraventricular hemorrhage. *Pediatric Critical Care Medicine.* 2006;7(1):40-44.
36. Tottman AC, Bloomfield FH, Cormack BE, Harding JE, Taylor J, Alsweiler JM. Sex-specific relationships between early nutrition and neurodevelopment in preterm infants. *Pediatric research.* 2020;87(5):872-878.
37. Walker M, Fitzgerald B, Keating S, Ray J, Windrim R, Kingdom J. Sex-specific basis of severe placental dysfunction leading to extreme preterm delivery. *Placenta.* 2012;33(7):568-571.
38. Wang L-W, Lin Y-C, Wang S-T, Huang C-C, Group TPIDCS. Identifying risk factors shared by bronchopulmonary dysplasia, severe retinopathy, and cystic periventricular leukomalacia in very preterm infants for targeted intervention. *Neonatology.* 2018;114(1):17-24.
39. Zhao D, Zou L, Lei X, Zhang Y. Gender differences in infant mortality and neonatal morbidity in mixed-gender twins. *Scientific reports.* 2017;7(1):1-6.

40. Zisk JL, Genen LH, Kirkby S, Webb D, Greenspan J, Dysart K. Do premature female infants really do better than their male counterparts? *American journal of perinatology.* 2011;28(03):241-246.
41. Zozaya C, Avila-Alvarez A, Arruza L, et al. The effect of morbidity and sex on postnatal growth of very preterm infants: a multicenter cohort study. *Neonatology.* 2019;115(4):348-354.
